# Metabolic Syndrome: From Molecular Mechanisms to Novel Therapies

**DOI:** 10.3390/ijms221810038

**Published:** 2021-09-17

**Authors:** Ali Abbas Rizvi, Anca Pantea Stoian, Manfredi Rizzo

**Affiliations:** 1 Division of Endocrinology, Metabolism, and Lipids, Department of Medicine, Emory University, Atlanta, GA 30322, USA; ali.abbas.rizvi@emory.edu; 2 Division of Endocrinology, Diabetes and Metabolism, University of South Carolina School of Medicine, Columbia, SC 29208, USA; manfredi.rizzo@unipa.it; 3 Department of Diabetes, Nutrition and Metabolic Diseases, Faculty of Medicine, Carol Davila University, 8 Eroii Sanitari, 059474 Bucharest, Romania; 4 Department of Health Promotion, Mother and Child Care, Internal Medicine and Medical Specialties (Promise), University of Palermo, 90133 Palermo, Italy

The metabolic syndrome (MetS) consists of a cluster of metabolic abnormalities including central obesity, insulin resistance, glucose intolerance, hypertension, and atherogenic dyslipidemia [1]; it is rapidly emerging as a global health problem that increases the risk of developing type 2 diabetes and cardiovascular diseases [2]. An early recognition using clinical parameters and inflammatory markers is imperative in order to reduce morbidity and possibly mortality too, attributable to the syndrome. In addition, a number of susceptibility genes and adipokines have been identified that are thought to play a role in the genetic etiology of MetS, thus paving the way to new molecular insights [3,4,5]. Knowledge of the etiopathogenic pathways could facilitate novel therapeutic approaches to managing and treating MetS. 

The link between MetS and diabetes and its complications, such as cardiovascular diseases, is a newly developing paradigm with the central point being early atherosclerosis and endovascular inflammation, where atherogenic dyslipidemia seems to have a critical role [6]. LDL seems to be pivotal for the formation and progression of atherosclerotic plaques; however, LDL are not homogenous particles, since they differ for many important pro-atherogenic properties, including metabolic behaviour, affinity to the LDL receptor and susceptibility to oxidation [7]. Indeed, small, dense LDL particles are those with stronger atherogenic potential, and recognized as an independent risk factor for cardiovascular diseases [8].

This highlights the clinical importance of both quality and quantity of LDL. Patients at higher cardiovascular risk have elevated concentrations of small, dense LDL [9,10,11,12,13], which are strictly linked to the early stages of subclinical atherosclerosis and endothelial dysfunction, both enhancing the risk of cardiovascular events [14]. Of interest, some novel anti-diabetic agents have shown favorable effects in subjects with the MetS [15] due to an improvement of patho-physiological alterations [16,17], and increasing evidence is accumulating for incretin-based therapies as effective measure for preventing of cardio-metabolic complications [18]. 

In the last 18 months the MetS has received particular attention, since diabetes, obesity, and hypertension have been shown to be significantly associated with an increased risk for more severe forms of COVID-19 and related deaths [19]. The optimal management to address the multiple components of cardiometabolic risk during COVID-19 is still evolving. It has been suggested that a thoughtful approach to the management of cardiometabolic disorders would reduce inflammation, improve immune response, and prevent deterioration in case of SARS-Cov-2 infection [20]. The data gathered thus far could guide future management of patients with the MetS in order to reduce the risk of developing these complications [21].

This Special Issue aims to provide an update on the latest research in MetS, shedding light on emerging markers, unravelling potential molecular mechanisms, and highlighting innovative remedies to be utilized in concert with lifestyle modifications. We hope that the issue will assist readers in keeping abreast of the health challenges and their emerging solutions in the modern era of the COVID-19 pandemic. Our intention is to gather relevant knowledge under one umbrella as a resource for clinicians and for the benefit of patients with the MetS and its complications. 
